# Electromagnetic Radiation Disturbed the Photosynthesis of *Microcystis aeruginosa* at the Proteomics Level

**DOI:** 10.1038/s41598-017-18953-z

**Published:** 2018-01-11

**Authors:** Chao Tang, Chuanjun Yang, Hui Yu, Shen Tian, Xiaomei Huang, Weiyi Wang, Peng Cai

**Affiliations:** 1 Physical Environment Group, Key Laboratory of Urban Environment and Health, Institute of Urban Environment, Chinese Academy of Sciences, 1799 Jimei Road, Xiamen, 361021 P.R. China; 2 University of the Chinese Academy of Sciences, 19 Yuquan Road, Beijing, 100049 P.R. China; 3 Xiamen Key Laboratory of Physical Environment, 1799 Jimei Road, Xiamen, 361021 P.R. China

## Abstract

Photosynthesis of *Microcystis aeruginosa* under Electromagnetic Radiation (1.8 GHz, 40 V/m) was studied by using the proteomics. A total of 30 differentially expressed proteins, including 15 up-regulated and 15 down-regulated proteins, were obtained in this study. The differentially expressed proteins were significantly enriched in the photosynthesis pathway, in which the protein expression levels of photosystems II cytochrome b559 α subunit, cytochrome C550, PsbY, and F-type ATP synthase (a, b) decreased. Our results indicated that electromagnetic radiation altered the photosynthesis-related protein expression levels, and aimed at the function of photosynthetic pigments, photosystems II potential activity, photosynthetic electron transport process, and photosynthetic phosphorylation process of *M. aeruginosa*. Based on the above evidence, that photoreaction system may be deduced as a target of electromagnetic radiation on the photosynthesis in cyanobacteria; the photoreaction system of cyanobacteria is a hypothetical “shared target effector” that responds to light and electromagnetic radiation; moreover, electromagnetic radiation does not act on the functional proteins themselves but their expression processes.

## Introduction

Electromagnetic radiation is an important environmental factor. On October 2, 2015, the United Nations Economic Commission for Europe (UNECE) issued a paper titled, “The UNECE–ITU Smart Sustainable City Indicators”^1^. In the environmental field, electromagnetic radiation was listed as a core indicator, together with solid waste disposal and perception of environmental quality. Electromagnetic radiation not only has a potential long-term effect and threat to public health but may also impact the ecological environment. In recent decades, researchers have carried numerous experiments to evaluate the biological and health effects of *in vitro* and *in vivo* exposure to non-ionizing radiofrequency fields in animals, humans and their isolated cells^2^. Plants were also used in studying the effects of EMF on living organisms, and electromagnetic irradiation induced different alterations in the enzyme activities^3^, and affected gene expression in plants^4,5^.

Photosynthesis is the most basic material and energy metabolism in the biosphere. Light is an electromagnetic wave with an electromagnetic radiation speed of 2.998 × 10^8^ m/s, which is the speed of light. The wavelength of each radiation is different, and the spectra required for photosynthesis reside in the visible range (380–750 nm). Quantity (duration and intensity) and quality (color or wavelength) of electromagnetic waves are key factors that are involved in regulating growth and physiological processes in photosynthetic organisms^6^. Han investigated the effects of different light colors on photosynthetic pigments of cyanobacteria, and found that red light (660 nm) alters both the amounts and proportions of phycocyanin and allophycocyanin; moreover, cyanobacteria responded to red light by regulating the composition and location of phycobilisomes^7^. Ultraviolet-A (320–400 nm) exposure significantly decreased the photosynthetic parameters of unicellular green alga^8^. Effects of the 300 MHz electromagnetic field were observed on the photosynthetic cells of tobacco, and results showed that the effects generated damage in the membrane of photosynthetic cells in the tobacco leaves, the barricade in the transmission process of electrons in photosynthesis, and decreased potential activity and photochemical efficiency of photosystems II^9^. Microwaves with low power density significantly influenced quantitative increase in the photosynthesis pigment levels of the vegetal cells^10^. Our previous study found that oxidative stress of *Microcystis aeruginosa* (*M. aeruginosa*) could be induced under electromagnetic radiation exposure, and regulations on key enzymes of photosynthesis (Rubisco and FBA) by electromagnetic radiation indicated that electromagnetic radiation could affect the photosynthesis of *M. aeruginosa* cells^11^.

The influence of Electromagnetic wave on living organisms has been observed in different animals and plants species. However, studies on the effects of electromagnetic radiation on photosynthesis have been few and mainly focused on the phenotypic level. Moreover, information is lacking regarding the mechanism of electromagnetic waves on photosynthesis. How electromagnetic radiation or photoelectromagnetic coupling affects photosynthesis and whether electromagnetic radiation and light have a homologous receptor or receptors should be investigated. We tried to observe the mechanism of electromagnetic radiation in photosynthesis at the proteomics level in this novelty study.

*M. aeruginosa* belongs to the order cyanophyta, which is the dominant population in many bloom-forming lakes, which occupy important niches in the environment and is a common habitat of algae with high environmental sensitivity. The most common and widely used mobile communication frequency in mainland China is 1.8 GHz. In this paper, the effects of 1.8 GHz and 40 V/m electromagnetic radiation on the photosynthetic system protein expression of algal cells were examined through proteomics to explore the mechanism of electromagnetic radiation on photosynthesis.

## Results

### Differential protein analysis

Between the treatment and control groups, 30 differentially expressed proteins were determined, 15 of which were up-regulated and 15 were down-regulated. The top 10 different protein expressions contained 4 uncharacterized proteins that were obtained and the molecular functions of the other 6 proteins were mainly related to chlorophyll binding, structural molecular activity, proton-transporting ATP synthase activity, and electron carrier activity. A detailed list of differential proteins is shown in Table 1.

**Table 1:** Differential protein list.

Name	Gene	Protein	CK	E	log2_FC (E/CK)	Pvalue
C789_1144	isiA	Iron stress-induced chlorophyll-binding protein	1.07 ± 0.07	1.57 ± 0.12	0.55661	0.00245
C789_1150	isiA	Iron stress-induced chlorophyll-binding protein	1.07 ± 0.07	1.57 ± 0.12	0.55661	0.00245
C789_1730	psbV	Cytochrome c-550	0.98 ± 0.03	0.78 ± 0.01	−0.32723	0.00047
C789_2057	C789_2057	Uncharacterized protein	0.96 ± 0.07	1.21 ± 0.14	0.331926	0.04327
C789_2058	coaD	Phosphopantetheineadenylyltransferase	1.01 ± 0.08	0.80 ± 0.07	−0.3328	0.02801
C789_2073	atpB	ATP synthase subunit a	1.04 ± 0.03	0.83 ± 0.08	−0.31787	0.01645
C789_2075	atpG	ATP synthase subunit b′	1.01 ± 0.01	0.71 ± 0.09	−0.50733	0.00680
C789_2296	C789_2296	NUDIX domain protein	1.01 ± 0.01	1.22 ± 0.06	0.281262	0.00259
C789_2366	speB	Agmatinase	0.94 ± 0.06	1.13 ± 0.03	0.264825	0.00981
C789_2954	ruvB	Holliday junction ATP-dependent DNA helicase RuvB	1.04 ± 0.04	0.86 ± 0.05	−0.26628	0.01180
C789_3280	C789_3280	Uncharacterized protein	1.02 ± 0.04	0.71 ± 0.15	−0.51681	0.04331
C789_4303	psbY	Photosystem II protein Y	1.00 ± 0.01	0.76 ± 0.06	−0.41025	0.00312
C789_428	C789_428	Uncharacterized protein	0.93 ± 0.06	1.17 ± 0.11	0.320073	0.03007
C789_4415	tpiA	Triosephosphate isomerase	0.99 ± 0.02	1.20 ± 0.03	0.282907	0.00040
C789_4588	gvpAII	Gas vesicle structural protein	1.02 ± 0.05	0.76 ± 0.08	−0.42609	0.01408
C789_4589	gvpAII	Gas vesicle structural protein	1.02 ± 0.05	0.76 ± 0.08	−0.42609	0.01408
C789_4839	C789_4839	Uncharacterized protein	1.01 ± 0.01	1.24 ± 0.03	0.301737	0.00018
C789_5042	hemC	Porphobilinogen deaminase	0.92 ± 0.10	1.12 ± 0.01	0.281214	0.03221
C789_53	C789_53	Uncharacterized protein	1.06 ± 0.05	0.74 ± 0.01	−0.51587	0.00034
C789_5304	psbE	Cytochrome b559 subunit alpha	1.01 ± 0.02	0.64 ± 0.12	−0.6609	0.01468
C789_549	C789_549	Uncharacterized protein	1.04 ± 0.04	0.84 ± 0.04	−0.31525	0.00225
C789_639	porB	S-layer domain protein	1.03 ± 0.03	1.35 ± 0.11	0.393678	0.00619
C789_83	C789_83	Uncharacterized protein	0.99 ± 0.05	1.96 ± 0.11	0.979031	8.70E-05
C789_820	C789_820	Uncharacterized protein	0.96 ± 0.03	1.19 ± 0.06	0.30699	0.00347
C789_844	gshB	Glutathione synthetase	0.96 ± 0.04	0.76 ± 0.10	−0.33466	0.04759
C789_87	C789_87	Uncharacterized protein	0.95 ± 0.06	1.61 ± 0.18	0.759683	0.00218
C789_895	C789_895	Uncharacterized protein	0.99 ± 0.05	0.81 ± 0.02	−0.2858	0.00383
C789_898	mdnF	Methyltransferase small domain protein	0.92 ± 0.13	1.23 ± 0.10	0.413078	0.04149
C789_RS03845			0.97 ± 0.03	1.26 ± 0.15	0.378397	0.01802
C789_1103	C789_1103	Uncharacterized protein	1.06 ± 0.06	0.80 ± 0.10	−0.39727	0.02415

### Differential protein GO enrichment analysis

The treatment group was compared with the control group in terms of cell composition analysis (Table 2). Differential proteins were mainly enriched in the cytoplasmic vesicle, protein complex, organelle membrane, membrane protein complex, proton-transporting two-sector ATPase complex, membrane-bounded organelle, and cytoplasmic vesicle. The cytoplasmic vesicle partial protein was the most enriched, and the number of differential protein was 2, which comprising 14.29% of the total differential protein; followed by protein complex-related protein, the number of differential protein was 7, which comprising 50% of the total differential protein.

**Table 2:** CKP-VS-EP GO Enrichment.

Number	GO ID	Description	GeneRatio (14)	BgRatio (447)	pvalue	qvalue
***Cellular Component***						
1	GO:0044433	cytoplasmic vesicle part	2 (14.29%)	2 (0.45%)	0.000913	0.015885
2	GO:0043234	protein complex	7 (50%)	70 (15.66%)	0.002392	0.015885
3	GO:0031090	organelle membrane	2 (14.29%)	3 (0.67%)	0.002690	0.015885
4	GO:0098796	membrane protein complex	5 (35.71%)	36 (8.05%)	0.003018	0.015885
5	GO:0033177	proton-transporting two-sector ATPase complex, proton-transporting domain	2 (14.29%)	4 (0.89%)	0.005282	0.018535
6	GO:0043227	membrane-bounded organelle	2 (14.29%)	4 (0.89%)	0.005282	0.018535
7	GO:0031410	cytoplasmic vesicle	2 (14.29%)	7 (1.57%)	0.017511	0.046081
8	GO:0031982	vesicle	2 (14.29%)	7 (1.57%)	0.017511	0.046081
***Molecular Function***						
1	GO:0046906	tetrapyrrole binding	4 (26.67%)	20 (1.59%)	0.000057	0.002934
***Biological Process***						
1	GO:0022900	electron transport chain	4 (26.67%)	20 (1.71%)	0.000076	0.008599
2	GO:0055114	oxidation-reduction process	4 (26.67%)	27 (2.31%)	0.000260	0.014764
3	GO:0006091	generation of precursor metabolites and energy	4 (26.67%)	39 (3.34%)	0.001110	0.042074

The second column is the GO term ID; the third column is the function description of the GO term; the fourth column is the number of differential genes that are noted to a GO term and the percentage of differential genes to the total number of differential genes (number of headings); the fifth is the number of genes annotated to a GO term and the percentage of genes to the total number of genes (number of headings); the sixth column is the P value; the seventh column is the Q value after multiple checks.

From the molecular functional analysis, 1.8 GHz radiofrequency radiation mainly had a significant effect on tetrapyrrole binding. Moreover, certain effects on the related proteins took place, such as monovalent inorganic cation transmembrane transporter activity, transition metal ion binding, inorganic cation transmembrane transporter activity, hydrolase activity, and adenylyltransferase activity. Four differential proteins related to tetrapyrrole binding existed, comprising 26.67% of the total differential gene.

From the biological process analysis, the most abundant GO protein of the differential protein was located in the electron transport chain, oxidation–reduction process, and generation of precursor metabolites and energy. The number of differential protein of each item was 4 and each comprised 26.67% of the total differential gene. Moreover, the differential genes were mainly associated with ATP biosynthesis and metabolic processes, nucleoside triphosphate metabolism and biosynthesis processes, nucleosides, nucleoside monophosphate, nucleotide biosynthesis and metabolism, glutathione metabolic process, protein-heme linkage, protein cofactor connections, hydrogen transport, glycosylated compounds synthetic processes, cell metabolism processes, biosynthesis, and metabolism.

### Analysis of Pathway Enrichment of Differential Proteins

**Figure 1:**
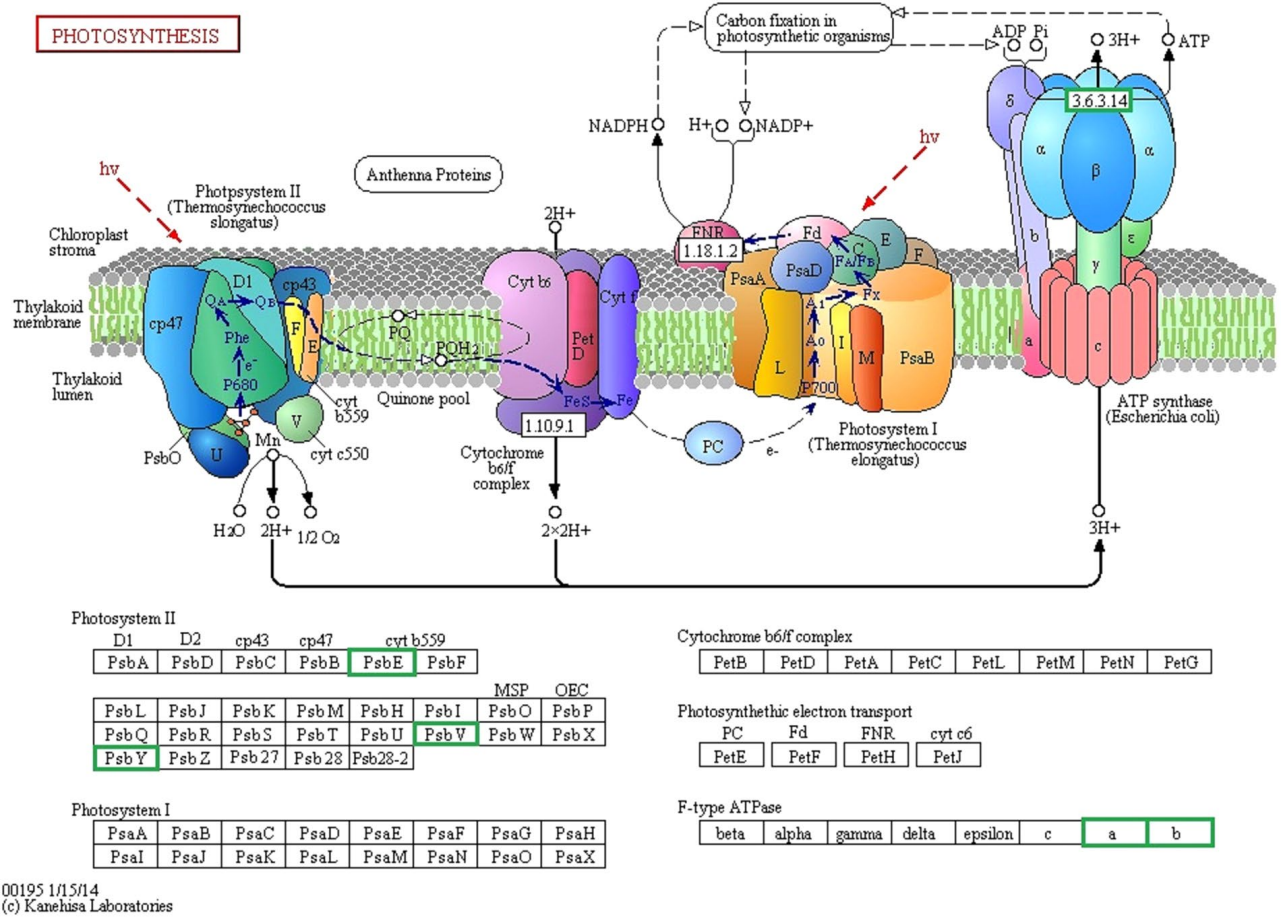
*M. aeruginosa* cells were exposed to radio frequency electromagnetic radiation and differential proteins mainly focused on the photosynthetic pathways, the significant differential proteins are marked by green box. As shown in Fig. 1, the expressions of cytochrome b559 subunit alpha, cytochrome c-550, protein Y, protein ATP synthase subunit a, and protein ATP synthase subunit b were down-regulated. This image is copyright permitted by KEGG^12–14^, http://www.kegg.jp/kegg/kegg1.html.

KEGG is a knowledge base for systematic analysis of gene functions, linking genomic information with higher order functional information, which is stored in the PATHWAY database (http://www.kegg.jp/kegg/kegg1.html). Algal cells were exposed to radio frequency electromagnetic radiation and differential proteins mainly focused on the photosynthetic pathways. Based on Fig. 1 of the photosynthesis in Kanehisa laboratories^12–14^, the significant differences in markers are shown in Fig. 1 and the corresponding differentially expressed proteins are listed in Table 3. Results showed that five differentially expressed proteins are related to photosynthesis, and the expressions of differential proteins were down-regulated after electromagnetic stress. As shown in Fig. 1, the expressions of cytochrome b559 (Cytb559) subunit alpha, cytochrome c-550, protein Y (PsbY), protein ATP synthase subunit a, and protein ATP synthase subunit b were down-regulated. Moreover, the differential proteins were mainly related to inositol phosphate metabolism, oxidative phosphorylation, pantothenate and CoA biosynthesis, homologous recombination, glutathione metabolism, fructose and mannose metabolism, arginine and proline metabolism, carbon fixation in photosynthetic organisms, cysteine and methionine metabolism, porphyrin and chlorophyll metabolism, glycolysis/gluconeogenesis, purine metabolism, carbon metabolism, and biosynthesis of amino acid function (Table 4).

**Table 3:** Differential proteins of Photosynthetic pathway.

Gene	Protein	log2(FC)
psbE(C789_5304)	Cytochrome b559 subunit alpha	−0.66
psbV(C789_1730)	Cytochrome c-550	−0.33
PsbY(C789_4303)	Photosystem II protein Y	−0.41
atpB(C789_2073)	Protein ATP synthase subunit a	−0.32
atpG(C789_2075)	Protein ATP synthase subunit b	−0.51

**Table 4:** Significant enrichment KEGG Pathway.

Number	Pathway	DEGs genes with pathway annotation (12)	All genes with pathway annotation (689)	Pvalue	Qvalue	Pathway ID
1	Photosynthesis	5 (41.67%)	47 (6.82%)	0.000660	0.009897	ko00195
2	Inositol phosphate metabolism	1 (8.33%)	3 (0.44%)	0.051418	0.385637	ko00562
3	Oxidative phosphorylation	2 (16.67%)	37 (5.37%)	0.131991	0.405275	ko00190
4	Pantothenate and CoA biosynthesis	1 (8.33%)	9 (1.31%)	0.147058	0.405275	ko00770
5	Homologous recombination	1 (8.33%)	9 (1.31%)	0.147058	0.405275	ko03440
6	Glutathione metabolism	1 (8.33%)	10 (1.45%)	0.162110	0.405275	ko00480
7	Fructose and mannose metabolism	1 (8.33%)	15 (2.18%)	0.233783	0.434740	ko00051
8	Arginine and proline metabolism	1 (8.33%)	16 (2.32%)	0.247425	0.434740	ko00330
9	Carbon fixation in photosynthetic organisms	1 (8.33%)	17 (2.47%)	0.260844	0.434740	ko00710
10	Cysteine and methionine metabolism	1 (8.33%)	23 (3.34%)	0.336857	0.505286	ko00270
11	Porphyrin and chlorophyll metabolism	1 (8.33%)	33 (4.79%)	0.447797	0.572373	ko00860
12	Glycolysis/Gluconeogenesis	1 (8.33%)	34 (4.93%)	0.457899	0.572373	ko00010
13	Purine metabolism	1 (8.33%)	41 (5.95%)	0.524000	0.604616	ko00230
14	Carbon metabolism	1 (8.33%)	71 (10.3%)	0.731840	0.784114	ko01200
15	Biosynthesis of amino acids	1 (8.33%)	94 (13.64%)	0.830597	0.830597	ko01230

## Discussion

During *M. aeruginosa* growth and development, changes in the environmental conditions, such as temperature, light, CO_2_, and nutrient composition, may induce changes in gene and protein expressions. Photosynthesis is the most basic material and energy metabolism of the biosphere and one of the most sensitive physiological processes for environmental change. Studies have shown that certain natural stresses (such as salt and sulfur deficiency) may reduce light-harvesting and photosynthetic activities, photosynthetic systems I and II, cytochrome b6/f, and ATP synthase gene expression levels. Low-temperature treatment, cytochrome a-b binding protein in the process of photosynthetic light absorption, cytochrome b559α, oxygen-enhanced protein, cytochrome b6-f complex Fe-S protein, and the photosystems I reaction center subunit protein showed significant down-regulation^15–17^. Electromagnetic waves also had certain effects on photosynthesis^10,11,18^. Under the action of a low level radio frequency electromagnetic field of 300 MHz, chlorophyll fluorescence dynamics process and ultra-weak photoemission in tobacco leaf underwent changes. Responses of the fluorescence dynamics parameters, such as F_0_, F_V_/F_0_, F_V_/F_m_, ΔF_V_/T, and T_1/2_, and the amount of ultra-weak photoemission to the radiating power of electromagnetic field appeared to be the characters of non-linear and power windows. Non-thermal effects of electromagnetic field were observed on the photosynthetic cells of tobacco. The effects generated damage in the membrane of photosynthetic cells in the tobacco leaf, the barricade of transmission process of electrons in photosynthesis, and the decrease in potential active and photochemical efficiency of photosystems II^10,18^. We investigated the proteomics of *M. aeruginosa* under electromagnetic radiation exposure and found that the differentially expressed proteins were significantly enriched in the photosynthetic pathway (Table 4).

In the exclusion of visible light in the environment, the expressions levels of Cytb559 α subunit, cytochrome c-550, and PsbY proteins were down-regulated in photosystems II of *M. aeruginosa* under electromagnetic radiation exposure (Fig. 1). The process of converting light energy into chemical energy by photosynthetic organisms was mainly catalyzed by four multi-subunit membrane-protein complexes: photosystem I (PSI), photosystem II (PSII), cytochrome b6f complex, and F-ATPase^19^. PSII mainly consists of D1, D2, CP43, CP47, Cytb559, other transmembrane proteins, several hydrophilic peripheral proteins, some small molecular weight protein subunits, and other components^20,21^. PSII is one of the most susceptible parts of the photosynthetic apparatus and is important in the photosynthetic response in higher plants to environmental perturbations and stresses^22^. Moreover, Cytb559 has redox activity that can produce photooxidation and photoreduction. It is a cytochrome molecule that binds to PSII and consists of two polypeptides (Mr4400 and 9300)0^23^. Cytb559 consists of two protein subunits (PsbE and PsbF) that ligate a heme-group between them. The exact function of this component in PSII has not yet been clarified, but its crucial role for the assembly and photo-protection in prokaryotic complexes has been suggested^24^. Furthermore, a functional role of Cytb559 in the protection of PSII under photoinhibition conditions *in vivo* has been determined^25^. Cytb559 plays an important role in the cyclic electron flow processes that protect PSII from light-induced damage during photoinhibitory conditions^26^. PsbY protein is important in controlling the redox of Cytb559; moreover, the PsbY protein Cytb559 is only present in its oxidized, and low potential form and plants with PsbY depletion are highly susceptible to photoinhibition^24^. Cytochrome c-550 plays a substantial role in maintaining the stability and function of the manganese cluster in algal PSII^27^. The absence of cytochrome c-550 hinders the balance of photosynthetic system under nitrogen-deficient conditions, which affects the nitrogen tolerance of bacteria^28^.

Expression levels of Cytb559 α subunit, cytochrome c-550, and PsbY protein in photosystem II of *M. aeruginosa* were down-regulated under electromagnetic radiation exposure, which indicated that electromagnetic radiation affects the abovementioned protein synthesis or translation, and this could affect the function of PSII Cytb559, the PSII cycle of electron flow and oxidation and reduction potential, and the function of cytochrome c-550. Therefore, the electromagnetic radiation will affect the light reaction processes of *M. aeruginosa*. In addition, the expression levels of two proteins in ATP synthase were down-regulated under electromagnetic radiation. ATP synthase on the thylakoid membrane, also known as H^+^-ATPase, catalyzed the synthesis process of ATP by ADP and Pi. The *p*H gradient of the thylakoid membrane, being the driving force of ATP synthesis and phosphorylation, can be blocked by killing venturicidin and similar reagents^23^. Plasma membrane H^+^-ATPase can function in the mitigation of physiological disturbances imposed by salt stress^29^. Electromagnetic radiation may affect the photosynthetic phosphorylation by affecting the ATP synthase on the thylakoid cells of *M. aeruginosa* and thus may affect photosynthetic phosphorylation. Electromagnetic radiation may be one of the reasons that indirectly affect photosynthetic carbon sequestration.

In conclusion, the response of the *M. aeruginosa* cells to electromagnetic radiation is a complex process, wherein differential expression is significantly enriched in photosynthetic pathways. In addition, the differential proteins are mainly related to inositol phosphate metabolism, oxidative phosphorylation, pantothenate and CoA biosynthesis, homologous recombination, glutathione metabolism, fructose and mannose metabolism, arginine and proline metabolism, carbon fixation in photosynthetic organisms, cysteine and methionine metabolism, porphyrin and chlorophyll metabolism, glycolysis/gluconeogenesis, purine metabolism, carbon metabolism, and biosynthesis of amino acid function. The PSII Cytb559 α subunit, cytochrome c-550, PsbY, and F-type ATP synthase (a, b) protein expression levels decreased. Electromagnetic radiation affects the photosynthesis-related protein synthesis or translation, and could affect the function of photosynthetic pigments, PS II potential activity, photosynthetic electron transport process, and photosynthetic phosphorylation process of *M. aeruginosa*.

Broadly speaking, visible light is within a certain wavelength range of electromagnetic wave (380–750 nm). Certain differences may exist in the electromagnetic radiation effect sites at different frequencies in the growth processes of *M. aeruginosa*. Visible light directly acts on the relevant proteins of photosynthetic system, but microwave electromagnetic radiation (1.8 GHz applied in this study) acts on protein synthesis or translation processes.

Based on the above evidence, the photoreaction system may be a target of electromagnetic radiation on the photosynthesis for cyanobacteria; the photoreaction system of cyanobacteria is a hypothetical “shared target effector” that responds to light and electromagnetic radiation; and electromagnetic radiation does not act on the functional proteins themselves but their expression processes. Furthermore, photosynthesis is associated with energy metabolism. Thus, the correlation between electromagnetic radiation and bioenergy metabolism must be further investigated.

## Methods

### Experimental Materials

The experiment involved species from *M. aeruginosa*, and the algal species came from the Institute of Aquatic Biology, Chinese Academy of Sciences, No. FACHB-905. Culture temperature was 25 ± 1 °C with 12 L:12 D light–to-dark ratio, the light intensity was 1000 ± 100 Lux, and the culture medium for high pressure sterilization was BG 11 medium.

### Experimental exposure device

**Figure 2:**
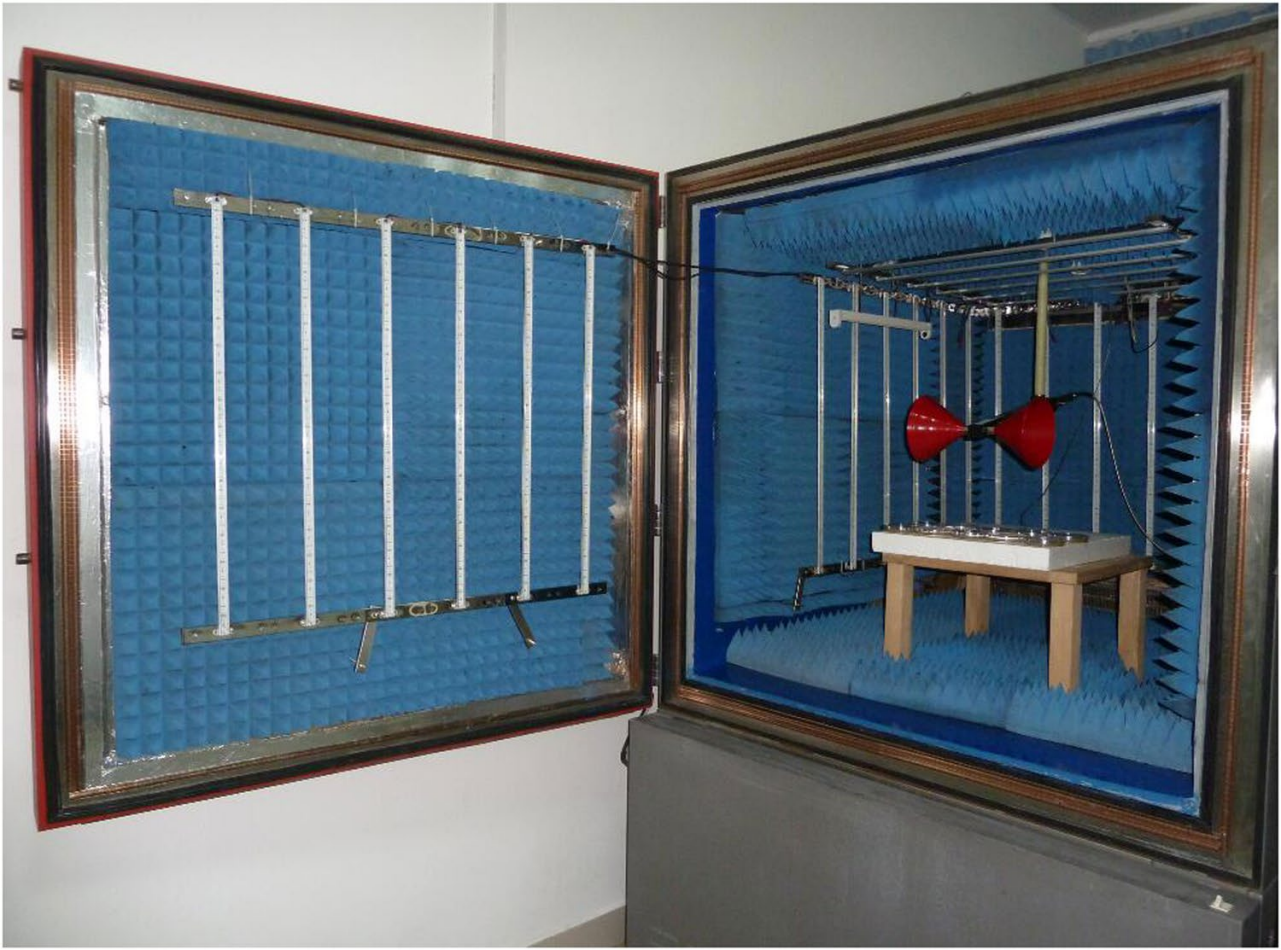
Radio frequency electromagnetic field exposure system.

Radio frequency electromagnetic field (RF-EMF) was generated using a vector signal generator (AgilentE8267DPSG, USA) and a signal amplifier (AV38701E, the 41st Institute of CETC, China). RF-EMF was emitted from an antenna (ETS3180B) that was placed at 24 cm above the sample area. A signal amplifier was used to amplify the RF/MW signal induced by the signal generator. The signal at the sample position was measured using an electromagnetic radiation analyzer (PMM8053B, Narta-STS, Italy) and a signal analyzer (AgilentN9030A). A series of operations was applied to ensure temperature accuracy and stability. First, the temperature probe of the incubator was placed adjacent to the sample so that the incubator would maintain its temperature according to that position. Second, the temperatures of the two incubators for the control and exposure samples were routinely calibrated using the same thermometer. Third, during the exposure periods, the temperature of the sample area was continuously monitored using temperature probes surrounding the control and exposure samples. Fourth, the surface temperature of each sample during the exposure period was checked using a thermal imager (Testo 890). All of the monitoring data showed that the temperature of the control and exposure samples remained stable^30^. Experimental exposure device was shown in Fig. 2. In this study, *M. aeruginosa* cells were exposed to 1.8 GHz RF-EMF through a continuous sine wave. At the position of the *M. aeruginosa* cells, the R F electromagnetic field strength was 40 V/m and the temperature was 25 °C.

### Experimental treatment

*M. aeruginosa* were cultured in the normal light for 30 days (Culture temperature was 25 ± 1 °C with 12 L:12 D light–to-dark ratio, light intensity was 1000 ± 100 Lux, and the culture medium for high pressure sterilization was BG 11 medium), and were then divided into two parts for exposure and control experiments.

To exclude the impact of light on the experiment, a dark condition was chosen for the experiment. The exposure group was treated with 1.8 GHz and 40 V/m electromagnetic radiation in the dark for 24 hours (based on our previous study result^11^), whereas the control group was not exposed to electromagnetic radiation and other conditions remained constant.

**Figure 3:**
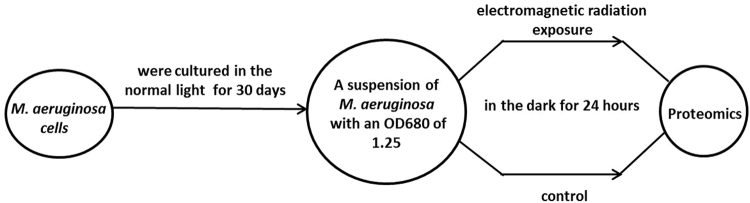
Experimental roadmap.

Experimental roadmap was shown in Fig. 3.

The above treatment was repeated thrice. The control mark was CK and the processing marker was E. Three samples each from the treatment and control groups were sent to GENE DENOVO Company to extract and identify proteins. The protein identification used Orbitrap Fusion Tribrid mass spectrometer by Gene Denovo Biotechnology Co. (Guangzhou, China).

### Data analysis

#### Database search

The mass spectrometry data were transformed into MGF files with Proteome Discovery 1.2 (Thermo, Pittsburgh, PA, USA) and analyzed using Mascot search engine (Matrix Science, London, UK; version 2.3.2). Mascot database was set up for protein identification using *M*. aeruginosa DIANCHI905 reference transcriptome. Mascot was searched with a fragment ion mass tolerance of 0.050 Da and a parent ion tolerance of 10.0 PPM.

#### Protein identification and quantification

The Mascot search results were averaged using medians and quantified. Proteins with fold change in a comparison >1.2 or <0.83 and unadjusted significance level p < 0.05 were considered differentially expressed.

#### Protein functional annotation and enrichment analysis

Proteins were annotated against GO, KEGG and COG/KOG database to obtain their functions. Significant GO functions and pathways were examined within differentially expressed proteins with p value ≤ 0.05.

### Data Availability

All data generated or analysed during this study are included in this published article.
